# Conditional *RAC1* knockout in motor neurons restores H-reflex rate-dependent depression after spinal cord injury

**DOI:** 10.1038/s41598-021-87476-5

**Published:** 2021-04-09

**Authors:** Curtis A. Benson, Kai-Lan Olson, Siraj Patwa, Marike L. Reimer, Lakshmi Bangalore, Myriam Hill, Stephen G. Waxman, Andrew M. Tan

**Affiliations:** 1 Department of Neurology, Yale University School of Medicine, 950 Campbell Avenue, Building 34, New Haven, CT 06510 USA; 2 Department of Veterans Affairs, Center for Neuroscience and Regeneration Research (127A), West Haven VA Medical Center, 950 Campbell Avenue, Building 34, West Haven, CT 06516 USA

**Keywords:** Spine regulation and structure, Spinal cord injury, Excitability

## Abstract

A major complication with spinal cord injury (SCI) is the development of spasticity, a clinical symptom of hyperexcitability within the spinal H-reflex pathway. We have previously demonstrated a common structural motif of dendritic spine dysgenesis associated with hyperexcitability disorders after injury or disease insults to the CNS. Here, we used an adeno-associated viral (AAV)-mediated Cre-Lox system to knockout Rac1 protein expression in motor neurons after SCI. Three weeks after AAV9-Cre delivery into the soleus/gastrocnemius of Rac1-“floxed” adult mice to retrogradely infect spinal alpha-motor neurons, we observed significant restoration of RDD and reduced H-reflex excitability in SCI animals. Additionally, viral-mediated Rac1 knockdown reduced presence of dendritic spine dysgenesis on motor neurons. In control SCI animals without Rac1 knockout, we continued to observe abnormal dendritic spine morphology associated with hyperexcitability disorder, including an increase in mature, mushroom dendritic spines, and an increase in overall spine length and spine head size. Taken together, our results demonstrate that viral-mediated disruption of Rac1 expression in ventral horn motor neurons can mitigate dendritic spine morphological correlates of neuronal hyperexcitability, and reverse hyperreflexia associated with spasticity after SCI. Finally, our findings provide evidence of a putative mechanistic relationship between motor neuron dendritic spine dysgenesis and SCI-induced spasticity.

## Introduction

Up to 80% of individuals with spinal cord injury (SCI) develop hyperreflexia and spasticity, which negatively affects quality of life and can impede rehabilitative efforts^1,2^. While currently available drugs, such as baclofen or Botox, have good utility in providing relief in the short-term, their long-term use carries the risk for complications, e.g., development of tolerance, systemic toxicity, and issues related with implanted infusion devices.

Spasticity is a clinical symptom of hyperexcitability within the spinal stretch reflex system, which presents as a velocity-dependent increase in tonic stretch reflexes with exaggerated tendon jerks^3^. Several central mechanisms may underlie pathological reflex control following injury or disease, including the loss of descending supraspinal and local segmental spinal inhibition, and maladaptive increases in intrinsic motor neuron excitability^4–7^. Additionally, injury-induced structural changes can powerfully affect reflex function associated with spasticity^8,9^

Dendritic spines are structural regulators of postsynaptic function, influencing the efficacy of synaptic transmission, and can change following disease or injury, including SCI^10–14^. Importantly, emerging evidence suggests that dendritic spine reorganization “locks-in” in the clinically intractable nature of SCI-induced spasticity^15–18^. Targeting molecular switches involved in dendritic spine dysgenesis may provide an avenue to alleviate spasticity. One such target is the Rac1 signaling pathway, which controls actin depolymerization through the downstream targets Pak1 and cofilin. Rac1 inhibits cofilin activity preventing actin depolymerization causing an increase dendritic spine stability^19,20^. In the context of SCI, disrupting Rac1 may attenuate dendritic spine dysgenesis by reducing the formation and maturation of dendritic spines. Treatment with a pharmacological Rac1 inhibitor can disrupt abnormal spine plasticity and partially restores spinal reflex function after SCI^21^. However, the use of pharmacological inhibitors has limited empirical utility due to non-specific tissue action.

To assess the specific role of Rac1-regulated dendritic spine plasticity in motor neurons in spasticity after SCI, we used a viral-mediated cre-loxp knockout system targeting Rac1. To retrogradely infect spinal alpha-motor neurons, we delivered AAV9-Cre into the soleus/gastrocnemius of Rac1-”floxed” adult mice. Three weeks after injection we observed a significant restoration of rate-dependent depression (RDD) and reduced H-reflex excitability in SCI animals. Viral-mediated Rac1 knockdown also reduced dendritic spine dysgenesis on motor neurons. In control SCI animals without Rac1 knockout, we continued to observe abnormal dendritic spine morphology associated with hyperexcitability disorder, including an increase in mature, mushroom dendritic spines, and an increase in overall spine length and spine head size. Collectively, these findings show that targeted disruption of Rac1 expression in ventral horn motor neurons can reduce dendritic spine dysgenesis, and partially reverse spasticity following SCI. This report provides further evidence for a novel mechanistic relationship between abnormal dendritic spine remodeling in the spinal cord motor reflex system and spasticity following traumatic injury.

## Materials and methods

### Animals

**Figure 1:**
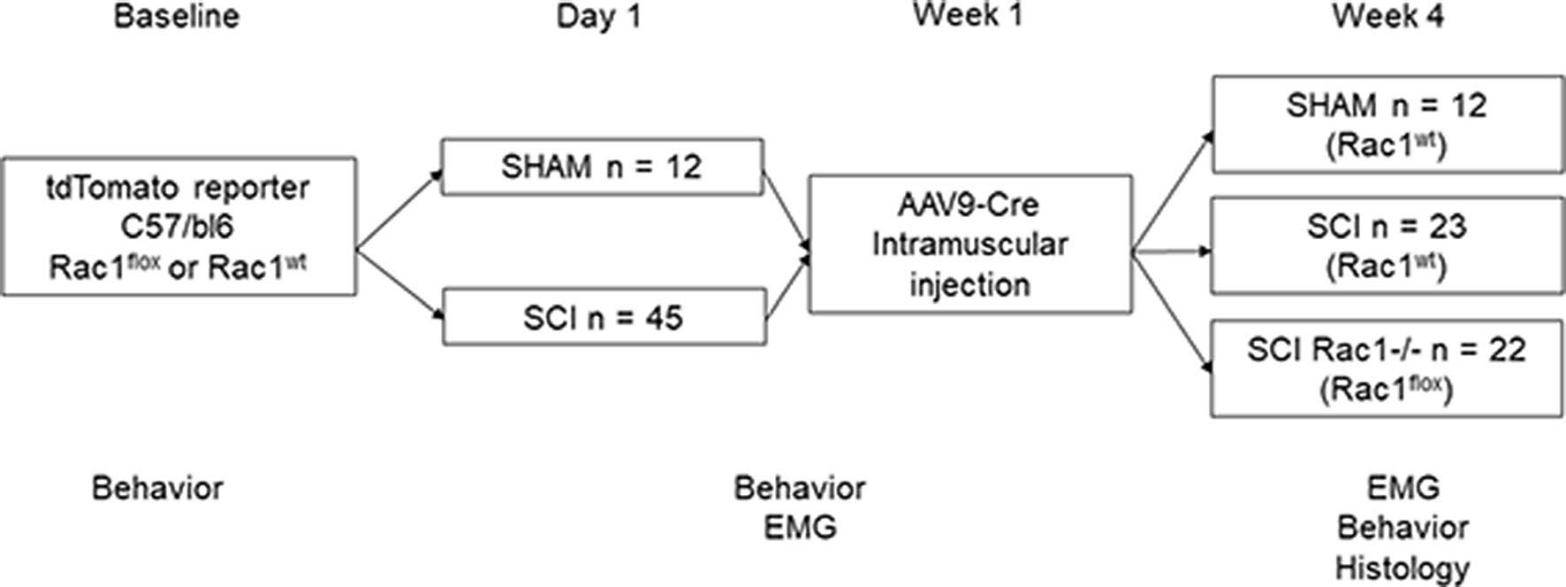
Study design. All animals underwent baseline behavioral testing and were randomly assigned to either SCI or sham groups. Spinal reflex excitability (EMG) and locomotor behavior was tested one-week post SCI or Sham prior to intramuscular injection of AAV9CMVCre into the left hindlimb soleus and gastrocnemius muscles. To assess the effect of conditional Rac1 KO (SCI Rac1−/−), locomotor behavior and spinal reflex testing was conducted after AAV9CMVCre injection at four weeks post-SCI. After completion of EMG and behavioral testing, tissue samples were collected for histology and dendritic spine analysis.

Experiments were performed in accordance with the National Institutes of Health *Guidelines for the Care and Use of Laboratory Animals* and in compliance with ARRIVE guidelines. All animal protocols were approved by the Yale University/Veterans Affairs Institutional Animal Use Committee. Animals were housed under a 12-h light–dark cycle with food and water provided ad libitum. Eight-week old male and female mice (c57/bl6) underwent either sham or SCI surgery. A total of 57 animals were used between sham (n = 12), SCI control (n = 23) and SCI Rac−/− (n = 22) groups (Fig. 1). Experimental animals were conditional tdTomato reporter Gt(ROSA)26Sortm9(CAG-tdTomato)Hze/J (Jackson labs, stock # 007909) crossed with Rac1^flox^ (Jackson labs, stock #005550). Control animals were littermates with conditional tdTomato expression and wildtype Rac1. The genotype of each animal was confirmed by PCR prior to study inclusion.

### Spinal cord injury

**Figure 2:**
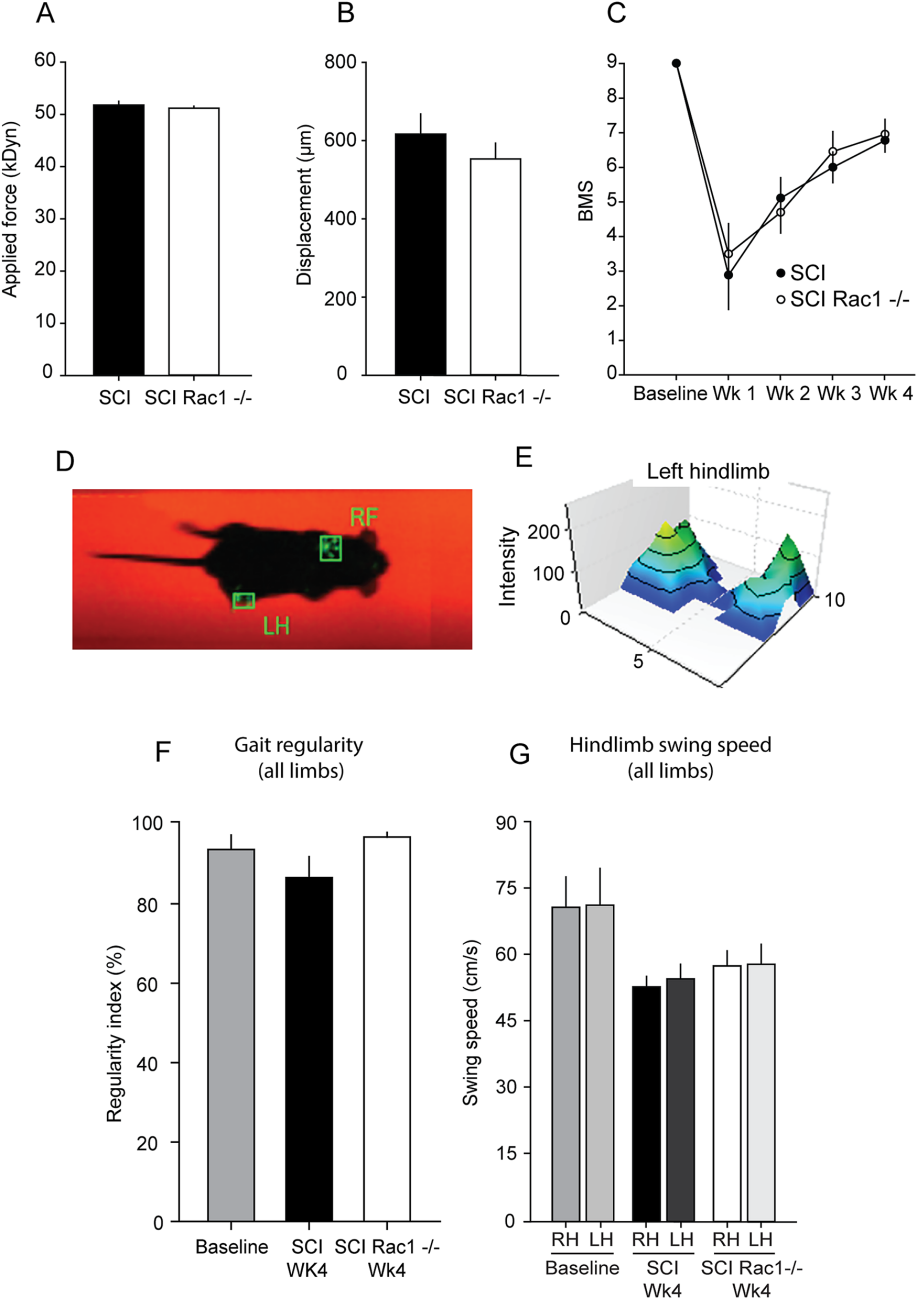
AAV9CMVCre mediated Rac1 KO does not impair recovery after SCI. (**A**, **B**) Biomechanical impact data provided by the Infinite Horizon impactor demonstrates that SCI Rac1−/− and SCI control groups received consistent injuries. (**C**) Blinded observers performed BMS testing at baseline, and weekly following SCI (Wk1-Wk4). Both SCI Rac1−/− and SCI control groups recovery equally over a 4-week period. (**D**) Representative video-still image of paw prints used for stepping pattern analysis acquired and analysis using the CatWalkXT (Noldus Information Technology). (**E**) Intensity plot of the left hind paw (virally injected side) acquired using the CatWalkXT (Noldus Information Technology) from an SCI animal four weeks post injury. (**F**) Four weeks after SCI, both SCI Rac1−/− and SCI control groups have a gait pattern (regularity index) similar to baseline testing. (**G**) Swing speed of the right (RH) and left (LH) hind limbs of SCI Rac1−/− and SCI group. Post SCI and viral injection there was no difference between hindlimb swing speed and compared to baseline. (* = p < 0.05). Graphs are mean ± SEM.

For animals with SCI, a laminectomy was performed at the 12th thoracic vertebra (T12) to expose the L2 spinal cord surface^22^. Briefly, mice were anesthetized with 1–3% isoflurane. A mild severity spinal contusion injury model was performed with an Infinite Horizons impactor (Precision Systems and Instrumentation, LLC)^23^. A metal rod (diameter: 1.3 mm) was applied to the exposed dorsal surface with a 50kDyn impact force. We measured biomechanical data for actual impact force and spinal cord displacement (μm) (see Fig. 2A, B). For Sham, animals underwent the same procedures, but without SCI. Following surgeries, muscle, fascia, and skin were closed with 6–0 monofilament sutures. Postoperative treatments included twice daily monitoring and post-operative injection of 0.9% saline solution (3.0 ml sc) and Baytril (0.3 ml, 3.5 mg/kg).

### Behavioral assays

To validate equivalent injury across SCI animals and assess whether AAV9CMVCre injection had an effect on overall locomotor ability, blinded investigators monitored animals using the Basso Mouse Scale (BMS)^24^. Each hindlimb was scored separately and averaged together per animal. The BMS grades locomotor function with 0 to 9 scores: 0 indicates complete paralysis of the hindlimbs; 9 indicates normal locomotor ability. Following baseline BMS measurements, animals with SCI were scored weekly and the average score in each week compared across groups. To further assess function, we used the CatWalkXT (Noldus Information Technology) system to assess kinematic function. Three runs across the walkway were averaged for each animal and compared across groups.

### Adeno-associated virus (AAV) intramuscular injection

Commercially available AAV9CMVCre (Iowa-1066) was purchased from the Gene Transfer Vector Core (lowa University). Intramuscular injections followed a previously described method^25^. Briefly, animals were anesthetized with isoflurane 1–3% and the left soleus and gastrocnemius muscle was exposed by a skin incision. Undiluted 2.5 × 10^13^ vg/ml AAV9CMVCre was injected into muscle tissue using a Hamilton syringe and 32-gauge 45-degree beveled needle. For optimal motor neuron infection yield, injections were repeated once daily for 3 days (total volume of 8–10 ul at 5 locations into the soleus/gastrocnemius tissue). Following injections, the skin incision was closed with 6–0 monofilament sutures.

### EMG H-reflex testing

We anesthetized animals with an induction dose of ketamine and xylazine (100/10 mg/kg, i.p.) and maintained on ketamine alone (20 mg/kg, i.p.)^26,27^. To record electromyogram (EMG) data, which included the muscle response (M wave) and the monosynaptic reflex (H reflex), we used a percutaneous needle preparation^5,28–31^. This minimally invasive procedure is analogous to methods used to study H-reflex in humans^31,32^. This method also permitted longitudinal study and maintained tissue integrity for histology at experimental endpoint.

For stimulation, a pair of Teflon insulated stainless steel wire electrodes (0.002″ diameter; A-M Systems, Inc., Carlsborg, WA) were threaded into a 32 G syringe needle. The wire ends were bent into sharp barbs. The insulation was removed with heat (exposed tips ~ 1 mm), and the needle/wire was then transcutaneously inserted with the tip in close proximity to the tibial nerve. The needle was retracted and the wire remained in place. A second electrode was inserted similarly ~ 2 mm away from the first electrode^21,26,27^. Stimulating electrode placement was adjusted until square wave stimulating pulses (0.2 ms duration given at a rate of 1 every 3 s) elicited visible motor twitch responses, i.e., plantar flexion^28,29^. To record EMG data (e.g., plantar reflex), an electrode was inserted into the plantar muscles within the hindpaw palmar/ventral surface, proximal to the ankle. A reference electrode was placed subcutaneously in the dorsolateral surface of the hind paw. These reflexes were chosen based on our pilot experiments and previous work that demonstrated EMG reflex response evoked in these muscles could be reproducibly recorded after SCI^33^. Moreover, plantar reflexes provide an established read out of reflexes elicited in other hind limb muscles, i.e., tibialis anterior and gastrocnemius, innervated by L4 and L5 nerves^28,29^. EMG responses were filtered, amplified, and analyzed offline using Spike 2 software (version 7.08; CED Software, Cambridge, UK). Threshold (T) was defined as the minimum intensity required for an M-wave response ~ 50% of the time. We used a stimulation intensity that produced consistent M- and H-wave responses (~ 1.4–1.8 T). To measure rate-dependent depression (RDD) of the H-reflex, we applied a paired-pulse stimulation paradigm: a control pulse and test pulse (0.2msec square) separated by a range of interpulse intervals (10–2000 ms). Three trials (10 sweeps/trial) were recorded for each interpulse interval. We quantified the M and H wave amplitudes from rectified and averaged waveforms^8,34^. For comparisons, the maximum amplitudes of the H and M response to the test pulse were converted into a percentage of the maximum amplitude response to the control pulse (test/control × 100). M and H waveforms were measured from baseline-to-peak amplitude. We calculated the H/M ratio using maximum amplitude of M-wave and H-reflex responses from the test pulse. All H-reflex testing was performed acutely (i.e., recordings lasted < 1 h per animal), and we ensured that all animals underwent similar H-reflex testing protocols.

### Immunohistochemistry and image analysis

At experimental endpoint, 4-weeks post-SCI/Sham surgery, all mice were euthanized under anesthesia (100/10 mg/kg, i.p.) via intracardial perfusion with ice cold phosphate buffered saline followed by 4% paraformaldehyde (PFA; 0.01 M PBS). Lumbar enlargement spinal cord tissue (L4-L5) were removed and post-fixed in 4% PFA at 4 °C overnight. For cryoprotection, we submerged tissue in 30% sucrose for ~ 48 h. 20 µm thick tissue sections were cut on a cryostat (Leica; Bannockburn, IL) and mounted on Superfrost + slides (Fisher Scientific; Pittsburg, PA). For immunohistochemistry, sections were blocked for 1 h at 25 °C in 4% normal donkey serum, 2% bovine serum albumin; 0.1% Triton-X100; 0.02%, 0.01 M PBS. Tissue was incubated in primary antibodies using our published protocols^34,35^ and included: rabbit anti-IBA1 1:500 (Abcam, ab178846), chicken anti-GFAP 1:500 (Abcam, ab4674), and rabbit anti-ChAT 1:200 (Millpore, AB143). Secondary antibodies included donkey anti-rabbit 647 (Jackson Labs, 711-496-152) and donkey anti-chicken 594 (Jackson Lab, 703-585-155). Immunofluorescent Z-stack images were captured with similar acquisition settings across groups using a confocal microscope (A1R, Nikon).

Tissue image analysis was conducted by blinded investigators using Image J software (National Institutes of Health free download: http://rsbweb.nih.gov/ij/). All AAV9CMVCre infected neurons expressed tdTomato-reporter. IBA1 and GFAP expression was analyzed from spinal cord tissue sections with tdTomato-reporter expressing motor neurons. Z-stack images were compiled into a single plane. Threshold-adjusted levels of IBA1 and GFAP immunoreactivity with subtracted background was compared across SCI groups as a percentage (%) of positive-pixel area to the total measured area of the ventral horn. AAV infection of lumbar motor neurons was confirmed by choline acetyltransferse (ChAT) immunolabeling^36,37^. To calculate the yield of viral-infected motor neurons in the ipsilateral injected and contralateral side, we measured the number tdTomato-expressing motor neurons co-labelled with ChAT. We report this data as a percentage (%) of the total number of ChAT-immunopositive motor neurons within the sampled spinal cord tissue sections (n = 10 tissue sections/side).

### Dendritic spine analysis

Investigators blind to treatment conditions performed all dendritic spine image analyses. Florescent tdTomato reporter expression in viral-infected motor neurons permitted the quantification of dendritic spines. Z-stack image volumes were acquired from tissue sections with an 0.2 microns step size using a Andor Dragonfly spinning disc confocal microscope (Andor Technology) using Andor iXon Ultra 888 electron multiplying charge coupled camera and Plan Apo Lambda 60 × (NA, 1.4) oil objective (Nikon Instruments). To identify α-motor neurons, we followed a screening workflow based on data from our previous study and others^21,33,34,37^. Alpha-motor neurons were identified based on their location in ventral horn Rexed lamina IX, had soma diameters > 25 μm, and cell body cross-sectional areas > 450 μm^2^^21^. As a refinement step for analysis a priori, we only included α-motor neurons for analysis that had a visible cell body and with greater than one dendritic branch. Three-dimensional (3D) traces of identified α-motor neurons were generated in Neurolucida 360 with the software’s user-guided tracing protocol (MicroBrightfield, Williston, VT). To ensure consistency, we traced dendritic branches visibly connected to the motor neuron cell body. To determine if there were any morphological differences across sampled neurons, we used Neuroexplorer (MicroBrightfield, Williston, VT) to measure cell body surface area, maximum and minimum cell body diameter, and the total dendritic branch lengths in each treatment group. We observed no differences in these morphological values across groups.

On 3D reconstructed α-motor neurons, we marked the location of dendritic spines on each dendritic branch. As previously described^21,38^, we defined a dendritic spine neck as the structure juxtaposed between the dendrite branch and the spine head base, which appears as a bulb-shaped structure. Thin- and mushroom-shaped spines were classified separately: thin spines had head diameters that were less than or equal to the length of the spine neck, whereas mushroom spines had spine head diameters that were greater than the length of the spine neck. Classification into only thin- and mushroom-spines allowed us to use simple, but strict rules in classifying spine morphology. Although this prevented measurement of subtle variations in spine shape, this method permitted collection of a large sample size. Moreover, the physiological impact of thin and mushroom spine shapes on neuronal and circuit physiology is well-described^10,16,39^. Dendritic spine density was expressed as spine number per μm of dendrite length. We used a Sholl analysis to assess changes in dendritic spine distribution relative to the cell body^15,21^. Dendritic spine density within proximal (0–40 μm), medial (40–80 μm), and distal (80–120 μm) regions from the cell body were averaged within each group and compared across groups. To quantify overall dendritic spine size, we measured spine length and head width. Spine length was defined as the distance from dendrite to the tip of the spine head. Whereas, spine head width was defined as the maximum diameter of the spine head.

### Statistical analysis

All statistical tests were performed at the α-level of significance of 0.05 by two-tailed analyses using parametric or nonparametric test, as appropriate. Normality assumptions of each data set was determined using a Shapiro–Wilk test. We used one-way ANOVA or Kruskal–Wallis one-way ANOVA on ranks followed by post hoc tests to correct for repeated measure error, i.e., Dunn or Bonferroni tests. Data management, statistical analyses and graph generation were performed using Sigmaplot 13.0 (Systat) and Microsoft Office Excel (2018). All data is described as mean ± SEM (graphs and text).

### Ethics approval

Experiments were performed in accordance with the National Institutes of Health *Guidelines for the Care and Use of Laboratory Animals* and in compliance with ARRIVE guidelines. All animal protocols were approved by the Yale University/Veterans Affairs Institutional Animal Use Committee.

### Consent for publication

All authors consent publication.

## Results

### Conditional Rac1 knockout does not impair gross locomotor recovery following mild SCI

To validate equivalent SCI severity across experimental groups (Fig. 1), we analyzed impact data from the Infinite Horizon device, i.e., *applied impact force* and *maximum spinal cord displacement* (Fig. 2A, B). Applied impact force predicts the amount of tissue sparing in this model, and correlates with locomotor outcome^40^. In SCI cohorts, we observed no difference in applied impact force across control SCI and experimental SCI (Rac−/−) groups (p > 0.05; SCI vs. SCI Rac−/−; 51.8 ± 0.7 vs. 51.2 ± 0.4 kDyn; ANOVA on ranks) (Fig. 2A). Similarly, we observed no difference in maximal spinal cord displacement (e.g., maximum depth of the impactor rod during contusion) between groups (p > 0.05; SCI vs. SCI Rac1−/−; 616.7 ± 50.8 vs 552.7 ± 40.6; one-way ANOVA) (Fig. 2B).

To determine whether AAV9CMVCre injection had an effect on locomotor ability, we used the BMS open-field testing paradigm^24^. Following baseline BMS measurements, SCI animals (from both wildtype or Rac1−/− groups) were scored weekly and the average score in each week compared across groups (Fig. 2C). In addition, the BMS score for the left and right hindlimbs were averaged for each animal, as there was no statistical difference between sides. One-week post-SCI, prior to injection of AAV9CMVCre, there was no difference in BMS scores between SCI groups with animal displaying ankle movement and some non-weight supported stepping (p > 0.05; SCI vs. SCI Rac1−/− 2.9 ± 1.0 vs 3.5 ± 0.9 BMS Score, Rank Sum Test) (Fig. 2C). Even after intramuscular injection of AAV9CMVCre, we also observed no effect on gross locomotor function four-weeks after SCI, with both groups able to perform coordinated stepping (p > 0.05 SCI vs SCI Rac1−/−; 6.8 ± 0.3 vs. 7.0 ± 0.4; one-way ANOVA). In addition, we analyzed the animals stepping pattern (Fig. 2D–G) using the CatWalk system. In CatWalk analyses four-weeks after SCI (Fig. 2D–G), we observed no difference in gait pattern and hindlimb swing speed, (p > 0.05; *regularity index*: baseline vs. SCI vs. SCI Rac1−/−, 94.4 ± 3.1 vs. 87.4 ± 4.8 vs. 97.6 ± 0.6; *hindlimb swing speed*: baseline RH vs. baseline LH vs. SCI RH vs. SCI LH vs. SCI Rac1−/− RH vs. SCI Rac1−/− LH, 71.2 ± 6.9 vs. 71.7 ± 8.3 vs. 53.2 ± 2.4 vs. 55.0 ± 3.3 vs. 58.0 ± 3.4 vs. 58.3 ± 4.5 cm/s: p > 0.05; one-way ANOVA with Bonferroni's post hoc test) (Fig. 2F, G). Indicating that AAV9CMVCre did not have a lateralized effect on gross motor function.

### Intramuscular AAV injection transfects ipsilateral motor neurons

**Figure 3:**
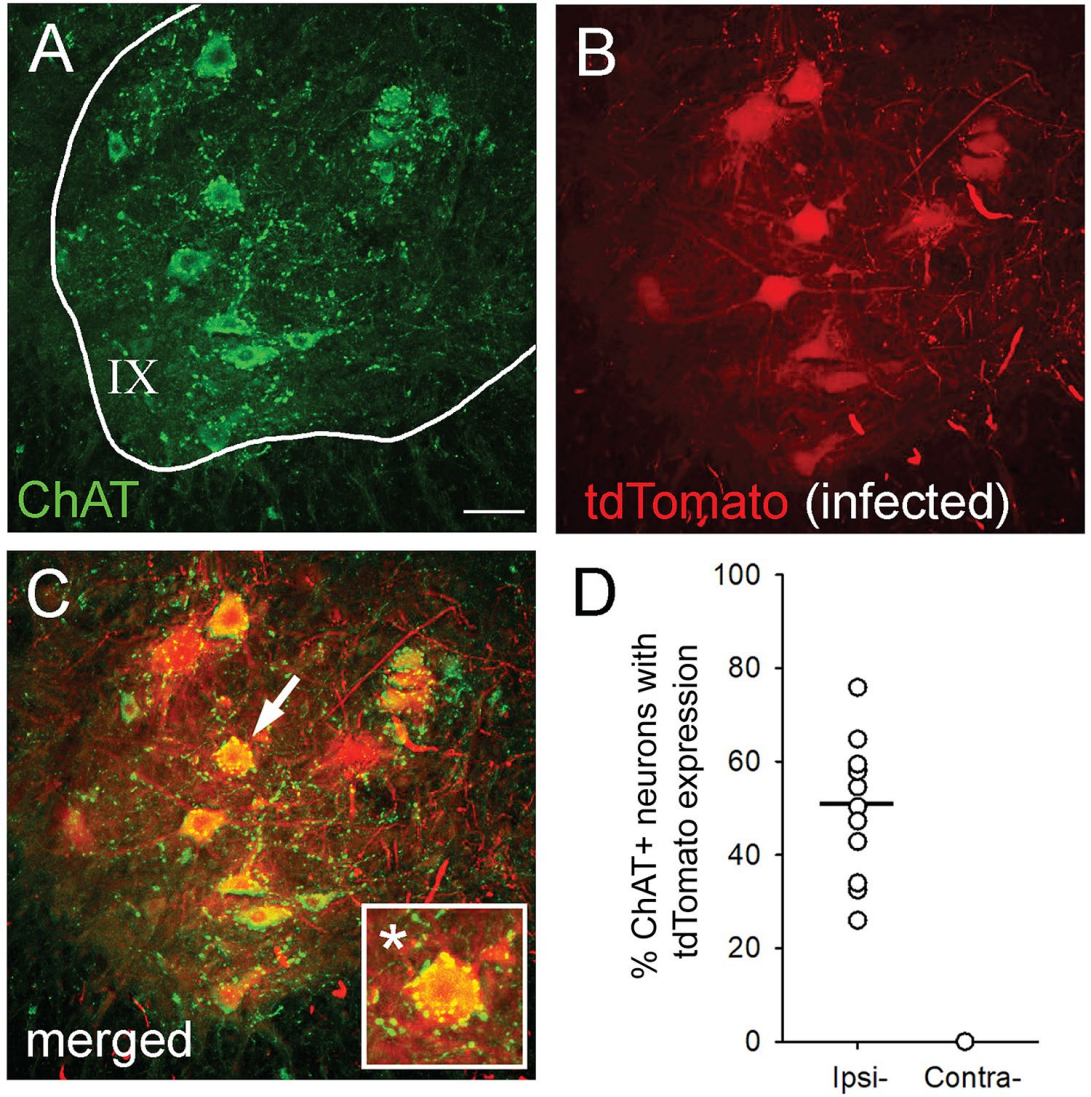
Intramuscular injection of AAV9CMVCre leads to expression of tdTomato in ventral spinal cord motor neurons. (**A**) ChAT positive motor neurons within lamina IX of the spinal cord. (**B**) AAV9CMVCre transfected motor neurons expressing tdTomato. (**C**) Merged image of (**A**) and (**B**) shows colocalization of ChAT positive neurons and tdTomato expression (arrow shows inset *). (**D**) The percentage of ChAT labelled motor neuron expressing tdTomato in the ventral horn of the spinal cord. AAV9CMVCre intramuscular injection tranfects 51.2% of ChAT-positive neurons in lamina IX on the ipsilateral injected side (Ipsi-). On the contralateral side (Contra-) there was no co-labelling between ChAT motor neurons and tdTomato expression. Data shown as scatterplot with mean. Scale bar in (**A**) = 50 µm and applies to images in (**B**) and (**C**).

To calculate infection yield of motor neurons, we measured the number tdTomato-expressing neurons co-labelled with ChAT within the ipsi- or contralateral spinal cord tissue sections sampled (n = 13 animals; 10 sections/animal) (Fig. 3). Three-weeks following intramuscular injections of AAV9CMVCre into the soleus/gastrocnemius tissue, we observed a transduction rate of 51.2% (range 24.7–77.6%) of ChAT positive ventral horn motor neurons in lamina IX (Fig. 3D). We did not observe any contralateral motor neurons expressing tdTomato reporter with ChAT co-labelling (Fig. 3D).

### Rac1 knockout in spinal motor neurons does not affect the glial inflammatory response

**Figure 4:**
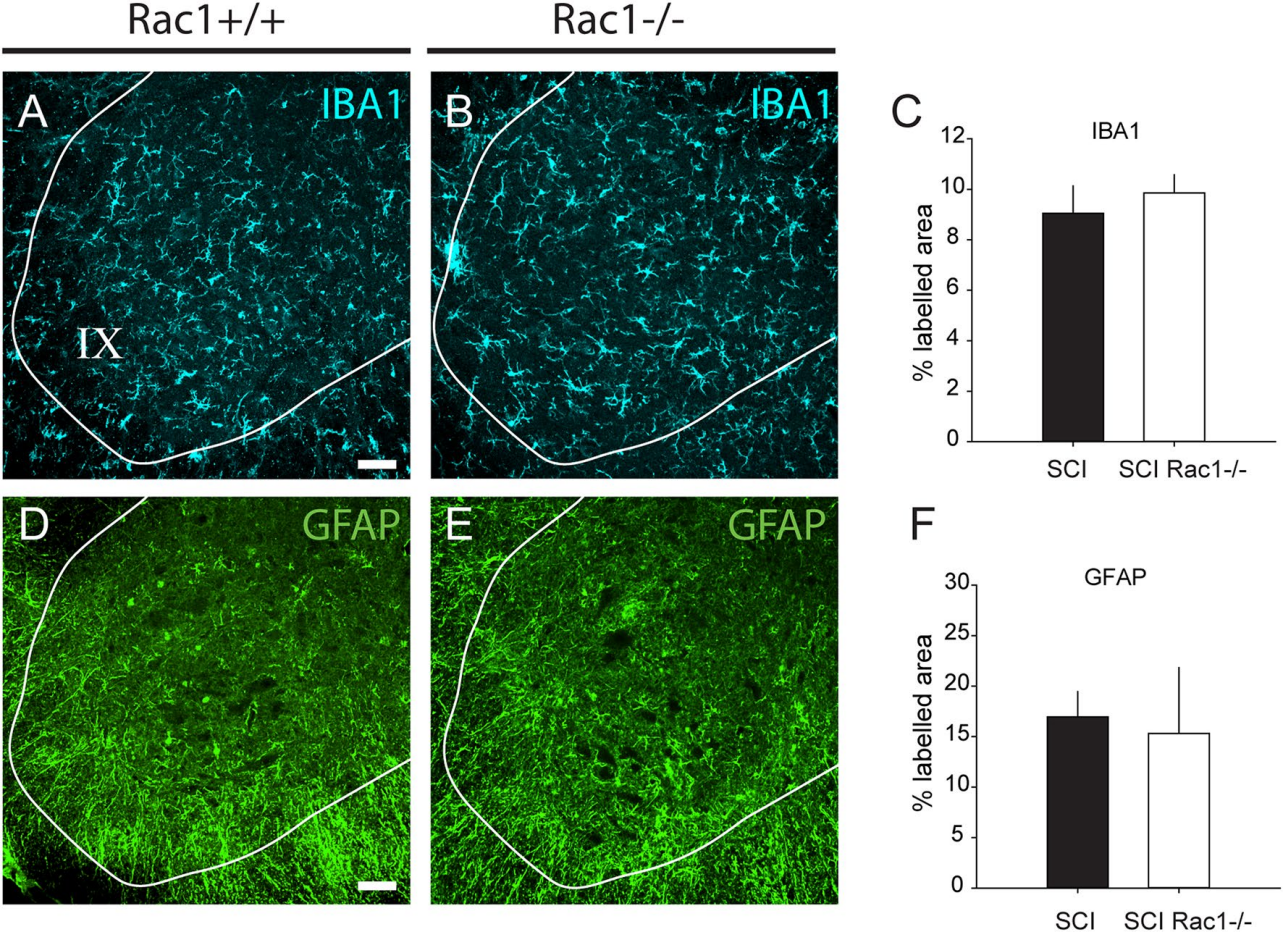
Conditional Rac1 KO in alpha-motor neurons did not alter microglia or astrocyte reactivity in the ventral horn. (**A**, **B**) IBA1 immunolabelling for microglia in the ventral horn of the spinal cord in SCI animals with (A) Rac1 or (B) Rac1 KO in alpha motor neurons. (**C**) No difference in the percent area labelled for IBA1 between SCI and SCI Rac1−/− groups. (**D**, **E**) Image of GFAP immunolabelling for astrocytes in the ventral horn of the spinal cord from animals with (**D**) Rac1 or (**E**) Rac1 KO in alpha motor neurons. (**F**) There was no difference in the percent area labelled for GFAP between SCI and SCI Rac1−/− groups. Graphs are Mean ± SEM; Scale bar in (**A**) and (**D**) = 50 µm and applies to images in (**B**), and (**E**).

To determine whether Rac1 KO affected microglia or astrocyte reactivity following injury, we measured expression levels of IBA1 and GFAP immunoreactivity, e.g., microglia or astrocyte markers, respectively, in the spinal cord ventral horn (Fig. 4A–F). We observed no difference in the percent area immunolabelled for IBA1 or GFAP between SCI controls and SCI with conditional Rac1 knockout in motor neurons (P > 0.05; IBA1: SCI vs. SCI Rac1−/−, 9.0 ± 0.7 vs. 9.0 ± 1.1% area, and GFAP, 15.3 ± 6.5 vs. 17.0 ± 5.6% area; one-way ANOVA) (Fig. 4C, F). Several studies have reported that *intravenously* administered AAV9 can infect astrocytes due to bioavailability through the vascular route^41,42^. However, in pilot studies we calibrated our AAV injection protocol to restrict viral exposure through low volume *intramuscular* injections^43^. As a result, we observed close-to-none GFAP-positive cells expressing tdTomato (data not shown). Similarly, in agreement with other work, we observed no overlap between tdTomato expression and IBA1, demonstrating that AAV9CMVCre did not have affinity for microglia^44,45^.

### Viral-mediated Rac1 knockout reduces H-reflex hyperexcitability after SCI

To determine whether Rac1 disruption could reduce hyperreflexia after SCI, we performed longitudinal EMG recordings to measure changes in evoked M- and H-reflex response. Under normal conditions, evoked H-reflex response exhibits rate-dependent depression (RDD), whereby increased activity reduces the overall reflex output. In diseases and injuries associated with spasticity, however, H-reflex exhibits a loss of RDD^8,26,29^. In SCI, reduced H-reflex RDD (physiologically measured through %H-reflex response and H/M ratio) is an electrophysiological and clinical diagnosis of spasticity^6,46–48^.

**Figure 5:**
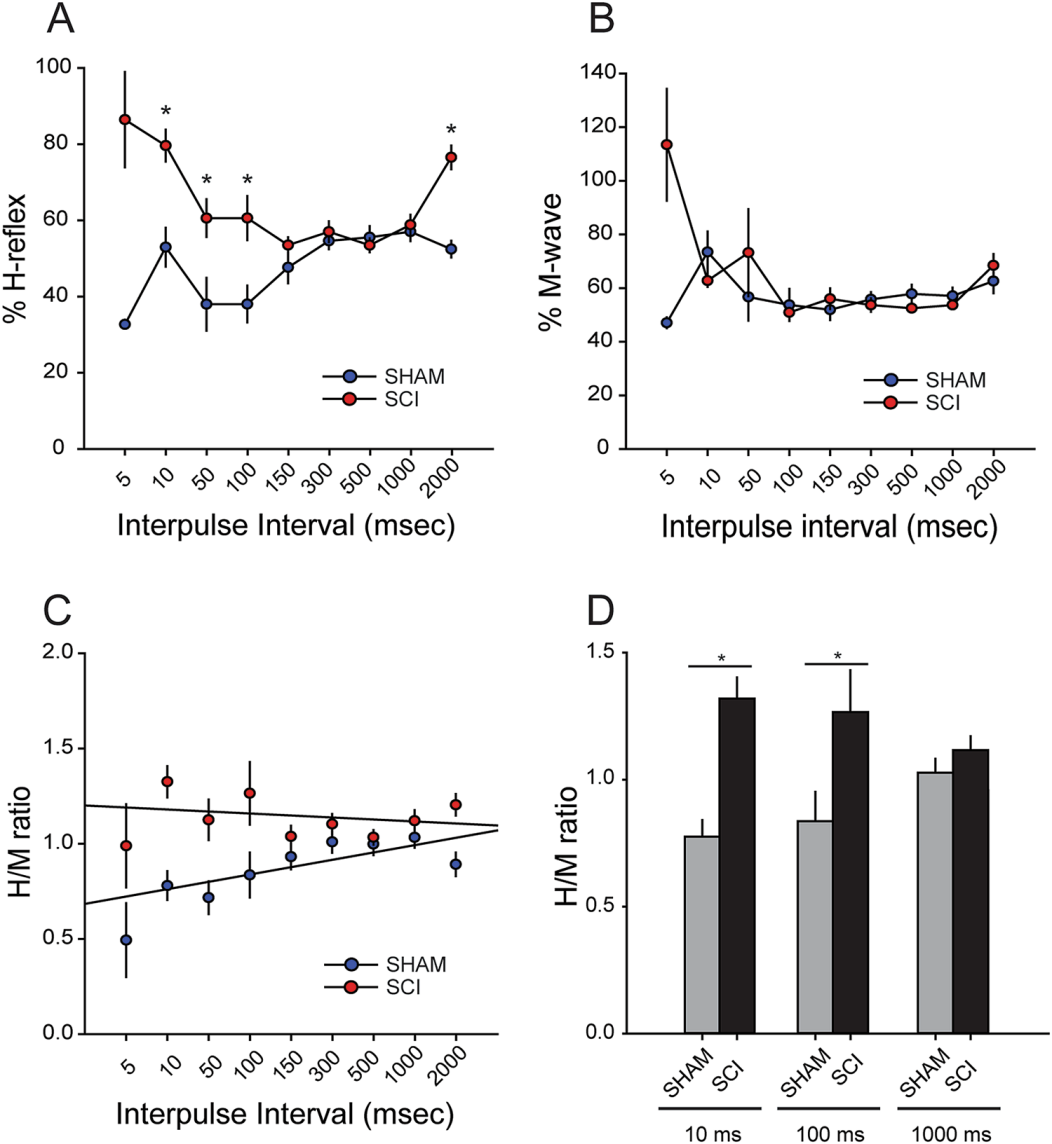
Enhanced H-reflex response 1-week after SCI. To confirm the development of hyperreflexia after SCI, we measured the H-reflex response using a paired-pulse paradigm (stimulating interpulse intervals 5–2000 ms). (**A**, **B**) % H-reflex and % M-wave are normalized values of the test pulse compared to the control pulse. (**A**) In Sham animals, shorter interpulse intervals are associated with less %H-reflex, demonstrating RDD. After SCI there was significant increase in the %H-reflex at 10, 50, 100 and 2000 ms interpulse intervals (* = p < 0.05). (**B**) There was no significant difference in %M-wave response between Sham and SCI. (**C**) H/M ratio was calculated by comparing peak amplitude of the test pulse evoked H- and M-wave response. H/M ratio in Sham produced a greater linear regression slope as compared to SCI, e.g., shallow slope. (**D**) SCI significantly increased the H/M ratio at 10 and 100 ms compared to Sham (* = p < 0.05). Graphs are mean ± SEM.

To measure H-reflex and M-wave responses, we stimulated the tibial branch of the sciatic nerve and recorded from the plantar muscle^21^. To elicit the H-reflex, we used a paired-pulse stimulation protocol consisting of a control and test pulse, separated by a range of interpulse intervals (5–2000 ms). To quantify evoked H-reflex response, we calculated the %H reflex in each group by measured and normalized the maximum amplitude of the H-reflex response (evoked from the test pulse) with the maximum amplitude of the H-reflex (evoked by the control pulse) (Fig. 5D).

One week after SCI or Sham, and before AAV9CMVCre injection (see design; Fig. 1), mice underwent EMG testing to confirm the presence of hyperreflexia, e.g., loss of RDD. As expected, SCI resulted in a loss in RDD compared to Sham (Fig. 5). We observed an increase in %H-reflex in SCI mice at 10, 50, 100, 2000 ms interpulse intervals compared to Sham, representing a loss of RDD and increased spinal reflex excitability (P < 0.05; sham vs. SCI at 10 ms 53.1 ± 5.1 vs. 79.8 ± 4.2%; at 50 ms: 38.1 ± 7.0 vs. 60.8 ± 5.0%; at 100 ms: 38.2 ± 4.9 vs. 60.8 ± 5.8%; 2000 ms: 52.6 ± 2.2 vs. 76.8 ± 3.2; one-way ANOVA with Bonferroni's post hoc test) (Fig. 5A). There was no difference in %M-wave at any interpulse interval between Sham and SCI animals 1 week after surgery (sham vs. SCI; at 5 ms: 32.8 vs. 86.7%; at 10 ms: 73.4 ± 7.7 vs. 62.8 ± 2.3; at 50 ms: 56.8 ± 8.8 vs. 73.2 ± 16.3; at 100 ms: 53.7 ± 6.0 vs. 50.9 ± 1.3; at 150 ms: 52.0 ± 3.8 vs. 56.0 ± 4.0; at 300 ms: 56.0 ± 2.7 vs. 53.7 ± 2.5; at 500 ms: 57.8 ± 3.4 vs. 52.5 ± 1.0; at 1000 ms 57.0 ± 3.1 vs. 53.7 ± 1.3; at 2000 ms 62.6 ± 4.5 vs. 68.4 ± 4.2; one-way ANOVA with Bonferroni's post hoc test)(Fig. 5B). Across the range of paired interpulse intervals, H/M ratio in Sham produced a steeper slope than compared with SCI animals (linear regression slope value, 0.04 vs. -0.01, respectively), demonstrating an increase in post-SCI reflex excitability (Fig. 5C). Additionally, SCI animals had a statistically greater H/M ratios at the 10 and 100 ms interpulse intervals (P < 0.05; Sham vs. SCI; 10 ms; 0.79 ± 0.08 vs. 1.32 ± 0.08; 100 ms: 0.8 ± 0.1 vs. 1.3 ± 0.2; 1000 ms: 1.03 ± 0.05 vs. 1.12 ± 0.06 H/M ratio; ANOVA on ranks) (Fig. 5C, D). Importantly, we did not observe differences in H-reflex function in SCI animals between either wild-type or “floxed” Rac1 animals (prior to AAV9CMVCre injection) (data not shown). This confirmed that transgenic “floxed” Rac1 mice develop a similar hyperreflexia profile to wild-type animals following SCI (i.e., in the absence of cre-recombinase expression).

**Figure 6:**
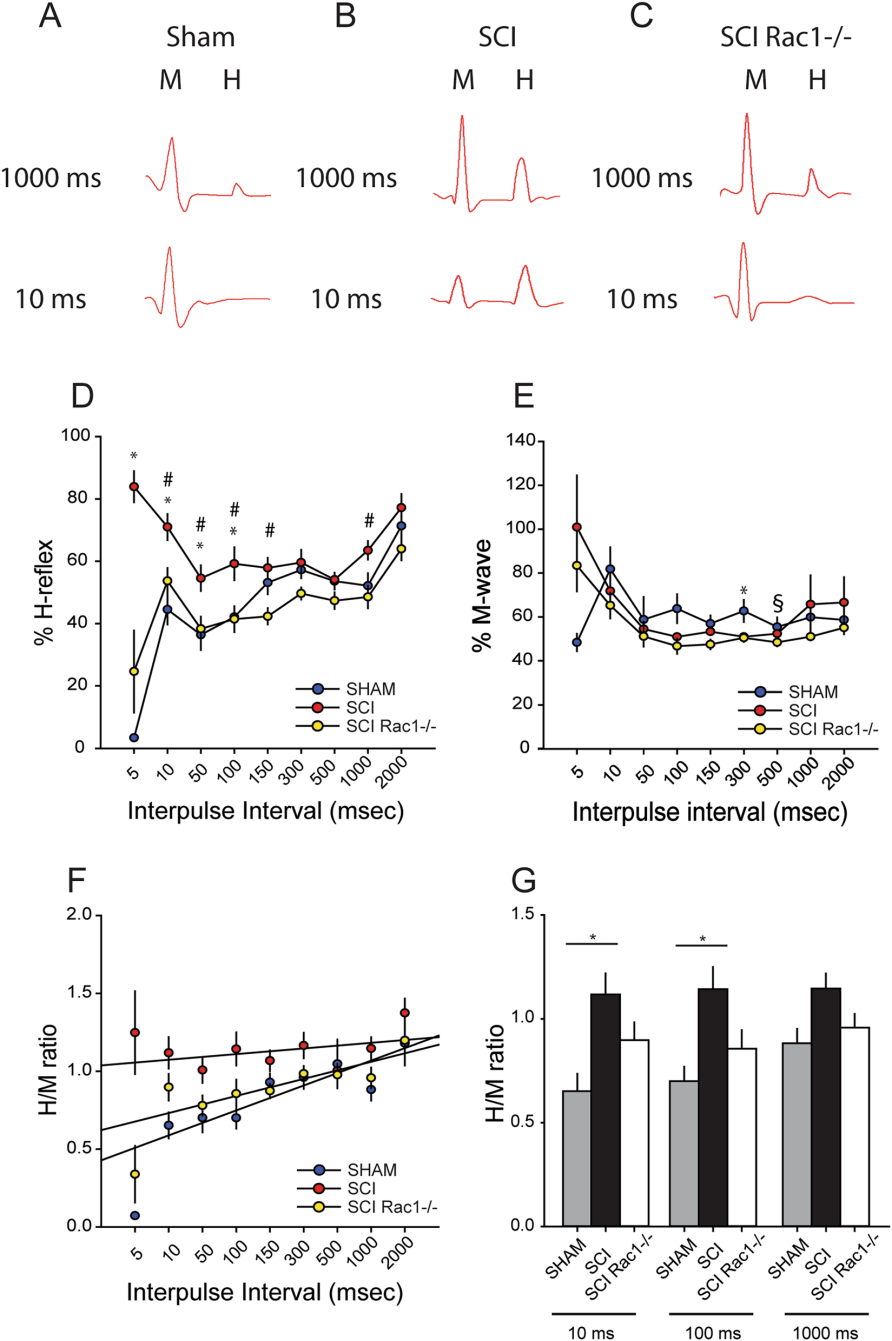
Disruption of Rac1 in alpha-motor neurons attenuates hyperreflexia after SCI. Representative test pulse traces of stim evoked H- and M-wave responses in (**A**) Sham, (**B**) SCI, and (**C**) SCI Rac1−/−. (**A**) Note that in Sham animals, as the interpulse intervals increases (10–1000 ms) between the test and control pulse, the amplitude of the H-response increase. (**B**) In contrast, SCI produces an exaggerated H-response at short (10 ms) interpulse intervals. (**C**) SCI Rac1−/− restores H-response depression at the short (10 ms) interpulse intervals. (**D**) After SCI there was a significant increase in %H-reflex at 5, 10, 50 and 100 ms interpulse intervals compared to Sham, indicating a loss of RDD (* = p < 0.05). SCI Rac1−/− animals had reduced %H-reflex at 10, 50, 100, 150 and 1000 ms interpulse intervals compared to control SCI animals, suggesting a restoration of RDD (# = p < 0.05). (**E**) %M-wave was mostly similar across the three groups with small, but significant differences between Sham and SCI at 300 ms interpulse interval (* = p < 0.05) and between Sham and SCI Rac1−/− at 500 ms interpulse interval (§ = p < 0.05). (**F**) SCI increased H/M ratio as demonstrated as stable linear trend line. Whereas, SCI Rac1−/− animals displayed a closer-to-normal H/M ratio, with a steeper linear trend line. G) SCI increased the H/M ratio at 10 and 100 ms compared to sham, indicating a loss of RDD (* = p < 0.05). Graphs are mean ± SEM.

To assess the effect of conditional Rac1 knockout in alpha-motor neurons, we tested H-reflex responses in the same animals at 3 weeks after intramuscular injection of AAV9CMVCre (Fig. 6). At this post-injection timepoint, control SCI animals continued to display an exaggerated %H-reflex at the shorter 5, 10, 50 and 100 ms interpulse intervals compared to Sham (Fig. 6D). In contrast, 3 weeks after AAV9CMVCre injection in mice with floxed Rac1, EMG recordings demonstrated closer-to-normal %H-reflex that were similar to Sham, indicating a partial restoration of RDD and attenuated hyperreflexia (p < 0.05; Sham vs. SCI vs. SCI Rac1−/−; 5 ms: 3.4 ± 0.4 vs. 83.9 ± 5.0 vs. 24.7 ± 13.1; 10 ms: 44.5 ± 4.0 vs. 71.0 ± 4.2 vs. 53.7 ± 4.1; 50 ms: 36.4 ± 4.9 vs. 54.6 ± 4.1 vs. 38.3 ± 4.0; 100 ms: 42.1 ± 2.3 vs. 59.2 ± 5.3 vs. 41.5 ± 4.2%; 150 ms: 53.2 ± 3.7 vs. 57.9 ± 3.3 vs. 42.3 ± 2.6; 1000 ms: 52.2 ± 4.0 vs. 63.5 ± 3.0 vs. 48.5 ± 3.6 H-reflex; one-way ANOVA with Bonferroni's post hoc) (Fig. 6D). %M-wave was similar across groups, with only minor differences at 300 ms and 500 ms interval (p < 0.05; Sham vs. SCI vs. SCI Rac1−/−; 300 ms: 62.8 ± 5.1 vs. 51.0 ± 1.2 vs. 50.5 ± 1.8; 500 ms: 55.5 ± 4.4 vs. 52.4 ± 1.1 vs. 48.4 ± 1.8%M-wave; ANOVA on ranks with Dunn’s post hoc) (Fig. 6E).

In measures of H/M ratio, we continued to observe the loss of RDD in control wildtype SCI animals 3-weeks post-AAV9CMVCre injection, as compared with Sham (linear regression slope, 0.05 vs 0.08, respectively) (Fig. 6F). In contrast, conditional Rac1 KO with AAV9CMVCre injection in SCI animals resulted in a sharper H/M ratio slope, similar to Sham (linear regression slope, 0.05). Specifically, the H/M ratio at 10, 100 and 1000 ms interpulse intervals revealed that SCI in wildtype controls resulted in a significant increase in H/M ratio that was partly restored by conditional Rac1 KO in spinal motor neurons (p < 0.05; Sham vs. SCI vs. SCI Rac1−/−; 10 ms: 0.7 ± 0.1 vs. 1.1 ± 0.1 vs. 0.9 ± 0.1; 100 ms: 0.7 ± 0.1 vs. 1.1 ± 0.1 vs. 0.9 ± 0.1; 1000 ms: 0.9 ± 0.1 vs. 1.1 ± 0.1 vs. 1.0 ± 0.1 H/M ratio; one-way ANOVA with Bonferroni's post hoc test) (Fig. 6F, G).

### Conditional Rac1 knockout reduces dendritic spine dysgenesis in alpha-motor neurons

Dendritic spine dysgenesis accompanies hyperexcitability disorders, e.g., pain and spasticity, as a result of injury and disease^17,21,49–51^. To assess the development of dendritic spine dysgenesis, we profiled dendritic spines in AAV9CMVCre-infected motor neurons expressing fluorescent tdTomato reporter. A total of 38 alpha-motor neurons were identified and sampled using inclusion criteria and reconstructed in the Neurolucida 360 environment (see Methods). To ensure equivalent sampling across treatment groups, we compared other cellular morphologies, which showed no differences across groups (p > 0.05; Sham vs. SCI vs. SCI Rac1−/−, *cell body area*: 580 ± 84.6 vs. 684.8 ± 70.6 vs. 544.4 ± 49.6 µm^2^; *cell body max diameter:* 39.1 ± 2.6 vs. 40.5 ± 3.0 vs. 35.8 ± 2.1 µm; *cell body min diameter:* 21.4 ± 2.3 vs. 25.8 ± 1.4 vs. 22.1 ± 1.1 µm; *total dendritic length*: 230.3 ± 17.0 vs. 373.2 ± 55.5 vs. 216.9 ± 34.6 µm; ANOVA on ranks with Dunn’s post hoc) (Table 1). Thus, any changes in dendritic spine morphology were likely not due to differences in sampling. At final endpoint, 4-weeks post-SCI or Sham surgery, a total of 1,518 dendritic spines were measured and analyzed by blinded investigators (Sham, n = 344 spines; SCI, n = 806; SCI Rac1−/−, n = 368 spines) (Table 1).

**Table 1:** Cell body dimensions and dendritic branch length of sampled tdTomato expressing spinal motor neurons.

	Cell body area (µm^2^)	Cell body max diameter (µm)	Cell body min diameter (µm)	Total dendritic diameter (µm)
Sham	580 ± 84.6	39.1 ± 2.6	21.4 ± 2.3	230.3 ± 17.0
SCI	684.8 ± 70.6	40.5 ± 3.0	25.8 ± 1.4	374.2 ± 55.5
SCI Rac1−/−	544.4 ± 49.6	35.8 ± 2.1	22.1 ± 1.1	216.9 ± 34.6

**Figure 7:**
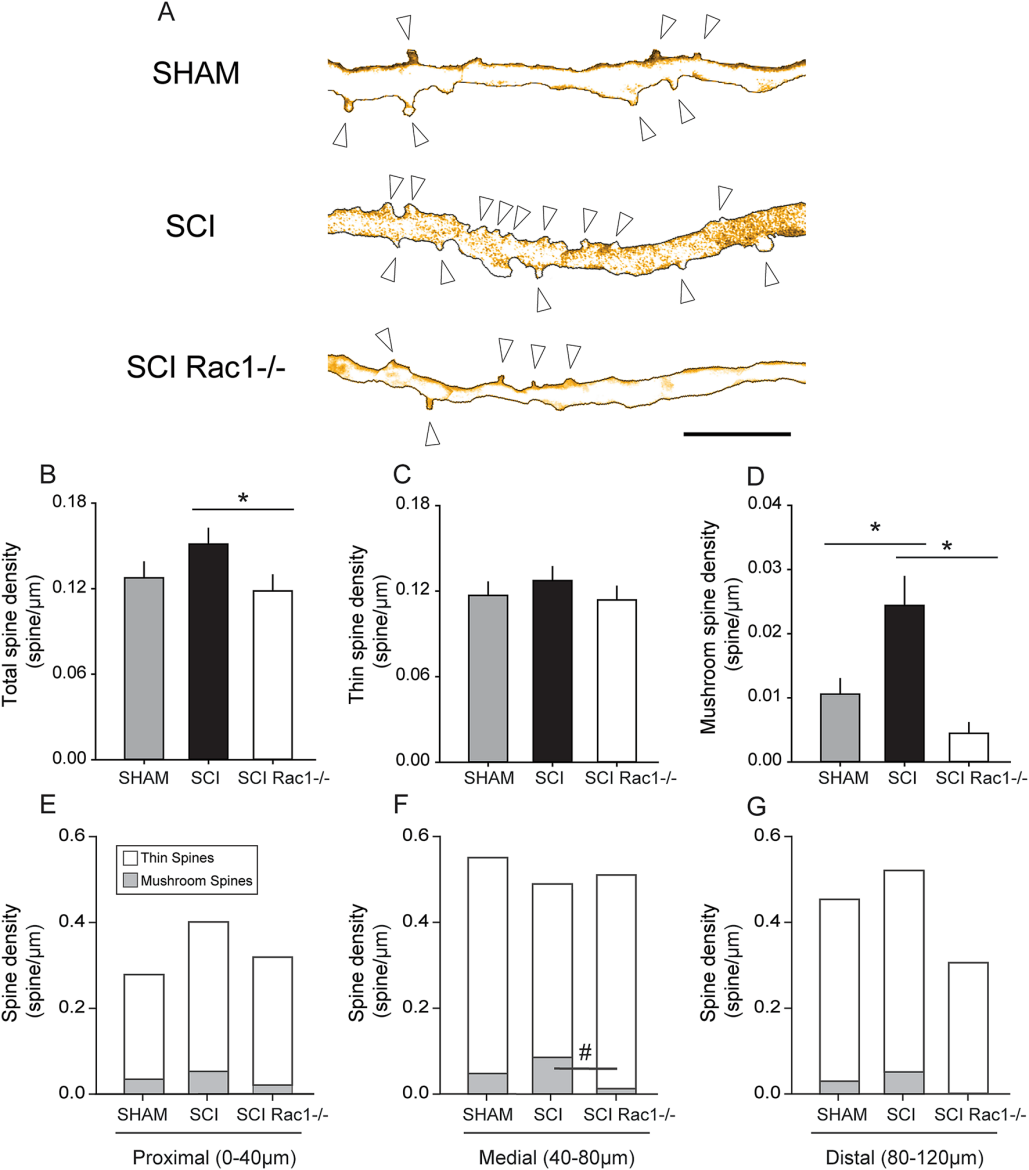
Conditional Rac1 KO in alpha-motor neurons reduces abnormal dendritic spine morphology associated with SCI and hyperreflexia. Analysis of dendritic spine profiles reveals differences in (**B**–**D**) spine density and (**E**–**F**) distribution. (**A**) Reconstructed dendritic segments from tdTomato filled spinal motor neurons showing apparent differences in dendritic spine profile between Sham, SCI and SCI Rac1−/− (arrow denotes spine). (**B**) Total spine density, which includes all spines, was significantly lower in the SCI Rac1−/− compared to control (* = p < 0.05). (**C**) There was no difference in the density of thin-shaped spines between groups. (**D**) SCI induced a significant increase in mushroom spine density compared to Sham (* = p < 0.05). In contrast, SCI Rac1−/− reduced mushroom spine density compared to SCI (* = p < 0.05). (**E**–**G**) Assessment of dendritic spine density within the (**E**) proximal (0–40 µm), (**F**) medial (40–80 µm) and (**G**) distal (80–120 µm) dendritic branches of tdTomato filled motor neurons. (**E**, **G**) Dendritic spine density was not different within the proximal or distal region between Sham, SCI and SCI Rac1−/−. (**F**) SCI induced an increase in mushroom spine density in the medial region, which was not observed in SCI Rac1−/− animals (# = p < 0.05). Graphs are mean ± SEM. Scale bar in (**A**) = 10 µm.

We analyzed the density of total-, thin- and mushroom-shape dendritic spines in alpha-motor neurons of SHAM, SCI and SCI Rac1−/− animals (Fig. 7A). Although SCI did not significantly change total spine density as compared with Sham (p > 0.05; Sham vs. SCI; 0.1 ± 0.01 vs. 0.2 ± 0.01 spines/µm; one-way ANOVA), viral-mediated conditional Rac1 KO in SCI animals significantly decreased total spine density in alpha-motor neurons (P < 0.05; SCI vs. Rac1−/−; 0.2 ± 0.01 vs. 0.1 ± 0.01 total spines/µm; one-way ANOVA with Dunn’s post hoc test) (Fig. 7B). We observed no change in thin-shaped spine density across any comparator group (p > 0.05; sham vs. SCI vs. Rac1−/−; 0.12 ± 0.01 vs. 0.13 ± 0.01 vs. 0.11 ± 0.01 thin spines/µm; one-way ANOVA) (Fig. 7C). Mushroom-shaped spine density significantly increased following SCI as compared with Sham or conditional Rac1 KO SCI animals (p < 0.05; Sham vs. SCI; 0.01 ± 0.002 vs. 0.02 ± 0.004 mushroom spines/µm; SCI vs. Rac1−/−: 0.02 ± 0.004 vs. 0.004 ± 0.002 mushroom spines/µm; one-way ANOVA with Dunn’s post hoc test) (Fig. 7D).

To investigate changes in the spatial distribution of dendritic spines, we used a Sholl analysis and compared spine densities at three distinct regional distances relative to the soma: proximal (0–40 µm), medial (40–80 µm) and distal (80–120 µm) (Fig. 7E–G). Here, we observed no difference in the spatial distribution of total spine densities (p > 0.05; total spine density, Sham vs. SCI vs. SCI Rac1−/−, *proximal*: 0.28 ± 0.05 vs. 0.40 ± 0.06 vs. 0.32 ± 0.04 total spines/µm; *medial*: 0.55 ± 0.07 vs. 0.48 ± 0.07 vs. 0.51 ± 0.06 total spines/µm; *distal*: 0.45 ± 0.1 vs. 0.52 ± 0.1 vs. 0.31 ± 0.08 total spine/µm; one-way ANOVA), or thin-shaped spine densities in the proximal, medial, or distal regions (p > 0.05; thin spine density; Sham vs. SCI vs. SCI Rac1−/−; *proximal*: 0.25 ± 0.05 vs. 0.35 ± 0.06 vs. 0.30 ± 0.03 thin spines/µm; *medial*: 0.50 ± 0.07 vs. 0.40 ± 0.07 vs. 0.50 ± 0.06 thin spines/µm; *distal*: 0.4 ± 0.1 vs. 0.4 ± 0.1 vs. 0.3 ± 0.1 thin spines/µm; one-way ANOVA) (Fig. 7E–G). Interestingly, mushroom-shaped spine densities increased in the medial region (i.e., 40–80 µm dendritic region relative to the soma) after SCI (Fig. 7F). Within this region, AAV9CMVCre infected alpha-motor neurons had significantly decreased mushroom spine density as compared to neurons in control SCI wildtype (p < 0.05; Sham vs. SCI vs. Rac1−/−, *medial*: 0.04 ± 0.01 vs. 0.08 ± 0.02 vs. 0.01 ± 0.01 mushroom spines/µm; ANOVA on ranks with Dunn’s post hoc) (Fig. 7F).

**Figure 8:**
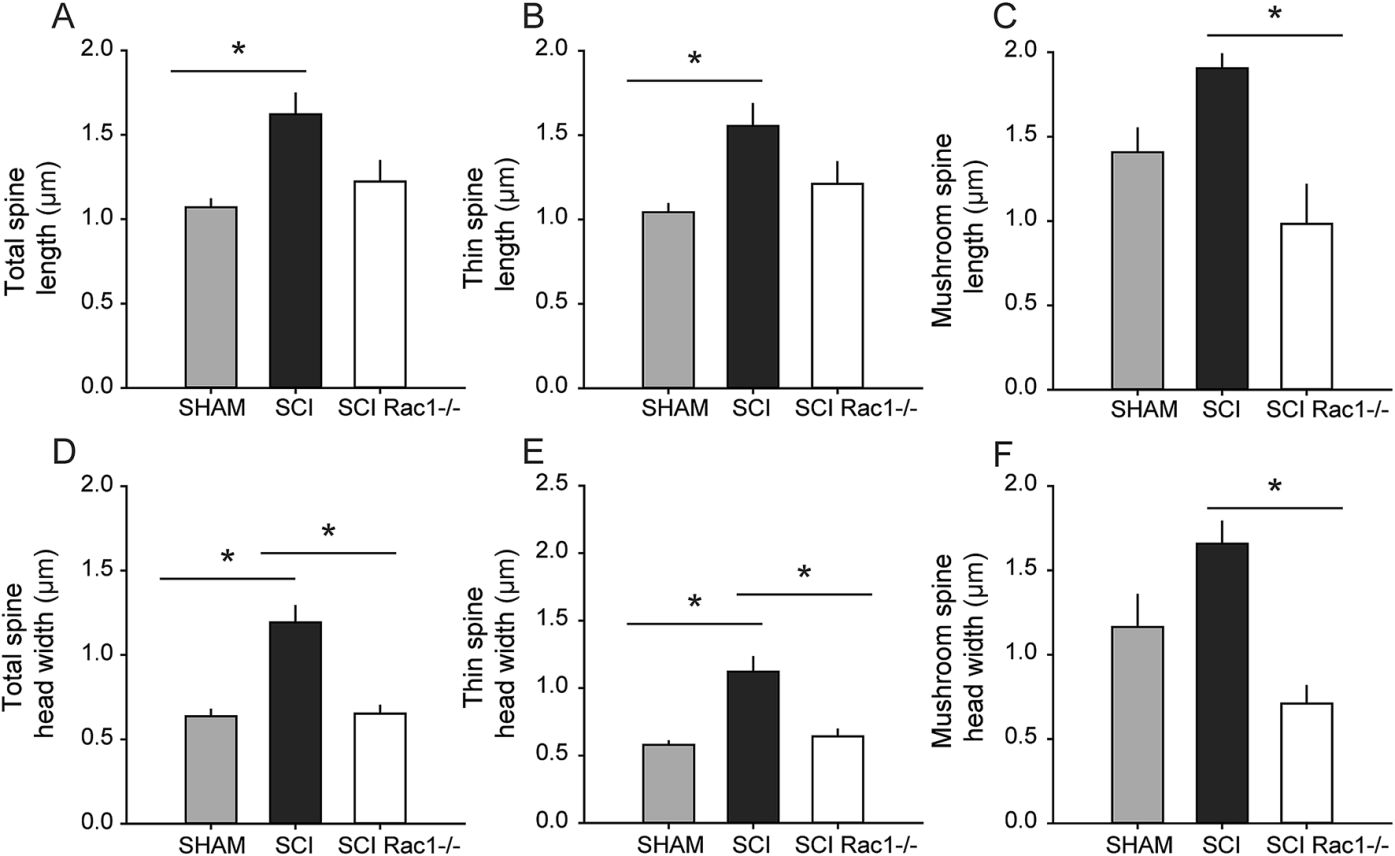
Conditional Rac1 KO normalizes dendritic shape on alpha-motor neurons after SCI. Analysis if dendritic spine (**A**–**C**) length and (**D**–**F**) spine head width. (**A**, **B**) SCI increased the length of total (all spines) and thin-shaped spine compared to Sham (* = p < 0.05). (**C**) Mushroom-shaped dendritic spines were significantly shorter in SCI Rac1−/− compared to control SCI (* = p < 0.05). (**D**, **E**) SCI resulted in a significant increase in spine head width for total and thin-shaped spines, which was not seen in the SCI Rac1−/− group (* = p < 0.05). (**F**) SCI Rac1−/− decreased the width mushroom-shaped spines compared to control SCI (* = p < 0.05). Graphs are mean ± SEM.

Dendritic spine size and shape regulate synaptic efficacy and can strongly influence neuronal excitability^16,52,53^. To determine whether conditional Rac1 KO could reverse SCI-induced changes in dendritic spine size, we measured spine length and head width of 1,518 spines from 38 neurons across Sham, SCI, and SCI Rac1−/− groups. As shown in Fig. 8, SCI increased the length of total- and thin-shaped spines, as compared with Sham (p < 0.05; Sham vs. SCI vs. SCI Rac1−/−, *total spines* 1.07 ± 0.05 vs. 1.6 ± 0.1 vs. 1.2 ± 0.1 µm; *thin spines*: 1.04 ± 0.05 vs. 1.6 ± 0.1 vs. 1.2 ± 0.1 µm; ANOVA on ranks with Dunn’s post hoc) (Fig. 8A, B). Conditional Rac1 KO resulted in no difference in the length of total- and thin-shaped spines as compared to Sham (Fig. 8A, B). On the other hands, conditional Rac1 KO significantly reduced in the length of mushroom-shaped spines as compared to control SCI (p < 0.05; Sham vs. SCI vs. SCI Rac1−/−, *mushroom spines*: 1.4 ± 0.1 vs. 1.9 ± 0.2 vs. 1.0 ± 0.2 µm; one-way ANOVA with Bonferroni post hoc) (Fig. 8C). In control SCI, spine head width for total- and thin-shaped spines increased compared to Sham (Fig. 8D, E). In contrast, conditional Rac1 KO in motor neurons decreased spine head width associated with SCI for total-spines, thin-, and mushroom-shaped spines (p < 0.05; Sham vs. SCI vs. SCI Rac1−/−, *total spines*: 0.64 ± 0.04 vs. 1.2 ± 0.1 vs. 0.65 ± 0.05 µm; *thin spines*: 0.58 ± 0.03 vs. 1.1 ± 0.1 vs. 0.64 ± 0.05 µm; *mushroom spines*: 1.2 ± 0.2 vs. 1.7 ± 0.1 vs. 0.7 ± 0.1; one-way ANOVA with Bonferroni post hoc) (Fig. 8 D-F).

## Discussion

Emerging evidence from our group and others has revealed a common structural motif of dendritic spine dysgenesis associated with neuronal hyperexcitability after SCI and nerve injury^14,15,21,54,55^. In nociceptive dorsal horn neurons, this motif is conserved in multiple models of pain^14,15,55–57^. In the present study, we report the first evidence demonstrating that viral-mediated conditional Rac1 knockout reduces dendritic spine dysgenesis in alpha-motor neurons and attenuates reflex hyperexcitability associated with spasticity after SCI.

Spasticity after SCI is a symptom of over-activity within the spinal stretch reflex circuit, e.g., H-reflex, and has been investigated in multiple SCI models^6,58^. To investigate the relationship between dendritic spine morphology and spasticity, we assessed the efficacy of disrupting Rac1 expression in spinal cord motor neurons after SCI. We performed a contusion SCI at spinal segment L2, a location that may replicate human lower thoracic-level SCI, and permits H-reflex testing of hindlimb musculature^1,6,59^. All animals with SCI presented with spasticity one-week after injury, exhibiting reduced RDD and increased evoked H-reflex responsiveness. Following viral-mediated expression of Cre recombinase in Rac1 “floxed” mice, we observed a reduction of hyperreflexia following SCI. Analysis of infected motor neurons expressing tdTomato reporter demonstrated that conditional Rac1 knockout also reduced overall dendritic spine density, specifically mushroom spine density, attenuated spine length and head width; and partially reversed abnormal spine distribution. Collectively, these anatomical findings demonstrate that Rac1 in spinal cord alpha-motor neurons contribute to dendritic spine dysgenesis associated with SCI-induced hyperreflexia.

Dendritic spine morphology is related to synaptic and neuronal activity and thus can provide a visual readout of neuronal function^11,60^. In healthy humans and rodents adaptive plasticity between sensory afferents and motor neurons can influence H-reflex function^46,61^, however after spinal cord injury maladaptive plasticity can contribute to uncontrolled H-reflex activity and spasticity^3,6^. In agreement, we observed SCI-induced changes in the density of thin and mushroom shaped dendritic spine on α-motor neurons four-weeks after injury, this was accompanied by loss of RDD and an increase in % H-reflex at short interpulse intervals. Importantly, SCI induced minimal changes in M-wave response, indicating that abnormal RDD and H/M ratios were largely due to mechanisms related to the dysfunction in the motor reflex monosynaptic circuit. Our data further confirms previous evidence in multiple disease models that dendritic spine dysgenesis can serve as a morphological correlate of dysfunctional neuronal activity^16,21,51,55,62^.

Our findings raise the question of how altered dendritic spine morphologies after SCI could directly contribute to hyperexcitable spinal reflex function. Interestingly, the monosynaptic spinal stretch reflex (i.e., H-reflex) operates through classical operant conditioning principles, e.g., a “memory engram” that regulates spinal reflex function^63–65^. In this context, the relationship between dendritic spine morphology and postsynaptic excitability has been well-studied in the field of learning and memory^66^. Following long-term potentiation (LTP) induction in hippocampal CA3–CA1 synapses, postsynaptic neurons exhibit an increase in dendritic spine number and volume. These late-phase LTP structural changes contribute to enhancing synaptic excitability through amplified excitatory glutamatergic transmission, e.g., more or larger spines improve postsynaptic AMPA receptor availability^67,68^. In the spinal cord, we observed an increase in more stable, mature spine profiles in alpha-motor neurons through an increase in mushroom-shaped spine density, as well as an increase in spine head size. As we and others have shown, more stable mushroom-shaped spine morphologies can have a greater impact on neuronal excitability as compared with thin-shaped spines^16,66^. Additionally, larger mature spines may contribute increased input discretization by narrowing EPSP waveforms, and increase synaptic transmission fidelity at higher rates of activity^69,70^. The added capability to transmit excitatory potentials at higher frequency with greater reliability could facilitate supra- and sub-threshold temporal summation^71–74^. Taken together, the biophysical properties associated with the dendritic spine changes we observed may explain the loss the H-reflex RDD and upshift in the H/M ratio associated with spasticity after injury.

This study selectively targeted Rac1 deletion in motor neurons via an intramuscular AAV-Cre injection route, e.g., reducing viral bioavailability to other CNS tissues. However, given that Rac1 contributes to regulating a multitude of intracellular molecular signaling pathways, we cannot entirely preclude off-target effects. Notably, Rac1 function is controlled by post-translational modification, such as prenylation and SUMOylation, which affect Rac1 localization to specific subcellular compartments^75,76^. In addition to actin cytoskeleton reorganization, Rac1 activity can also influence the spinal excitability through altered gene expression, production of reactive oxygen species (ROS), e.g., which can influence synaptic transmission, and inflammatory responses^77–79^. Thus, in our present study, Rac1 disruption could have *indirectly* affected reflex excitability along with structural changes to dendritic spine morphology. This does raise a related question as to whether these cellular reactions to injury act synergistically. For example, inhibition of Rac1 in diabetic focal cerebral ischemia led to neuroprotection, through reduced ROS and fewer Bcl-2/Rac1 mitochondrial complexes^79^. Suffice it to say, our work provides a compelling opportunity to further investigate the link between Rac1 activity and motor neuron excitability after SCI.

Following SCI, we observed an increase in spine size and density. These changes represent a common motif of dendritic spine dysgenesis associated with a neuronal hyperexcitability, in this case, the symptom of spasticity^14,55^. Multiple factors contribute to spine dysgenesis after SCI: inflammation, increased glutamate, abnormal glia response^21,80,81^. By targeting Rac1, we can attenuate abnormal spine remodeling regardless of the upstream mechanism. Rac1 activity is well known to control the formation and maintenance of dendritic spines. For example, in vitro experiments show that expression a dominant-negative form of Rac1 results in spine elimination, while constitutively active Rac1 increases spine density^82,83^. This fits with our observation that the deletion of Rac1 in motor neurons reduces the total- and mushroom spine density compared to SCI controls. In addition, Rac1 deletion reduced the size of both thin and mushroom shaped spines after SCI. This effect may be related to the role of Rac1 in actin cytoskeleton rearrangement: Rac1 regulates the actin nucleator actin-related protein 2/3 (Arp2/3) to drive Arp2/3 through the WAVE protein complex, which is necessary for activity dependent spine growth^84,85^. Finally, reduced spine size may be related to the role of Rac1 in LTP. For example, the LTP induction leads to subsequent Rac1 activation and dendritic spine growth^86^. Rac1 KO in motor neurons thus may inhibit maladaptive synaptic plasticity, e.g., LTP^18^, and abnormal spine morphology thereby reduce evoked reflex excitability following SCI.

This study provides the first reported evidence demonstrating that a viral-mediated conditional knockout model can significantly affect motor neuron dendritic spine structure in the injured spinal cord. We targeted Rac1 in spinal cord motor neurons using a conditional *cre-loxp* system with transgenic “floxed” Rac1 mice and intramuscular injection of AAV9CMVCre. Rac1 activity controls actin stability involved in the formation and stabilization of dendritic spine structure through the Rac1/Cdc42-PAK pathway^82,87–89^. In our study, a minimally invasive AAV injections into hindlimb muscle restricted infection to the ipsilateral innervating motor neurons and did not appear to affect non-neuronal cells, e.g., astrocytes or microglia, in the ventral horn^43^. This is in line with previous studies showing that AAV vectors can selectively infect neuronal tissue without damage to the CNS or contribute to chronic inflammation, e.g., low immunogenicity^90–93^. Importantly, viral-mediated Rac1 *cre-loxp* knockout in approximately 50% of L4 ventral horn alpha-motor neurons was sufficient to partially restore normal evoked H-reflex response after SCI. Although it is possible that viral injection procedures led to sensitization of sensory afferents within the spinal reflex pathway, we observed no difference across SCI groups in CatWalk sensory-motor outcomes. Although we have shown that intrathecally administrated doses of a pharmacological Rac1 inhibitor, NSC23766, can reduce H-reflex excitability in SCI animals with spasticity^21^, small molecule inhibitors have limited empirical and clinical utility. Taken together, our findings provide a unique basis for future studies aimed at developing a translational gene therapy by targeting the Rac1 pathway, perhaps as others have similarly done with virally-delivered miRNA knockdown constructs driven with motor neuron specific promoters^94,95^.

In summary, this study demonstrates that viral-mediated conditional Rac1 disruption in ventral horn motor neurons reduces abnormal dendritic spine dysgenesis and attenuates hyperreflexia associated with spasticity after SCI. Combined with our previous work^17,21,49,51^, these findings further support the emerging principle that dendritic spine dysgenesis is a morphological correlate of spinal cord hyperexcitability disorder, and provides an opportunity to explore strategies, including gene therapy approaches targeting dendritic spine dysgenesis, to correct abnormal reflex function after spinal cord injury.

## Data Availability

Data is available upon written request to Dr. Andrew Tan.
